# Production, pricing, and incentives in the fetal bovine serum market

**DOI:** 10.1093/tas/txag044

**Published:** 2026-04-10

**Authors:** Angela N Jungbluth, B Wade Brorsen, Derrell S Peel

**Affiliations:** Department of Agricultural Economics, Oklahoma State University, Stillwater, OK, 74078, United States; Department of Agricultural Economics, Oklahoma State University, Stillwater, OK, 74078, United States; Department of Agricultural Economics, Oklahoma State University, Stillwater, OK, 74078, United States

**Keywords:** animal welfare, byproduct, cows, hedonic pricing, market inefficiency

## Abstract

Fetal bovine serum (FBS) is a valuable byproduct of cattle production, critical for vaccines, cell therapy, and other cell growth applications. Despite its value, cattle producers are not given incentives to increase FBS supply. This paper describes the FBS production process and investigates the economic and institutional barriers to increasing FBS production. The marketing channels and production process are described by interviews with industry employees. Opinions from these interviews led us to the hypothesis that concerns about the public’s reaction to the animal welfare implications in the production of FBS are the primary cause for the market inefficiency of FBS. Production budgets show revenue potential and industry costs. To identify existing market incentives, least squares mean regression are used on replacement and slaughter cow auction price data. Results from the budget analysis show a positive net revenue from a pregnant cow, yet the results of the least squares regression show no significant incentives to produce a pregnant cow. This suggests that pregnant cows are not incentivized, regardless of the positive returns available, which aligns with interview responses that state animal welfare concerns stop purposeful production of pregnant cows for FBS production. While pregnant cows receive slightly higher prices, the premiums pale in comparison to the potential value of FBS. The budgets show potential profits of $908 per cow that is found to have a 23-kg fetus, but a maximum of $56.47 increase in premium for a cow that is further along in the pregnancy (7–9 months pregnant vs. 1–3 months pregnant) and would produce more serum. The current lack of incentives could be problematic for the future of FBS supply based on current short-term trends in the cattle markets.

## Teaser

Fetal bovine serum (FBS) is a valuable byproduct of cattle production, critical for vaccines, cell therapy, and other cell growth applications, yet it is only produced incidentally. While demand is high for FBS, direct incentives don’t exist in this market, but they may be necessary given current short-term trends in the cattle markets.

## Introduction

Fetal bovine serum (FBS) is categorized as inedible offal by the United States Department of Agriculture (USDA). When a pregnant cow is identified at a processing facility, blood is removed from the fetal calf, then processed further to remove blood cells and other impurities leaving just the raw serum. Serum can be valuable for scientific purposes as it is used as a component in cell culture growth that produces human vaccines and other cell culture uses such as cell therapy (Brindley et al. 2012). Many recent *Journal of Animal Science* used FBS; for example, see Li et al. (2024), Gresse et al. (2024), McClenahan (2025) or Swanson et al. (2025). FBS has been a major component in cultured meat production (Lee et al. 2023), but it is too costly to be a long-term solution (Garrison et al. 2022). FBS, although costly, is more effective for most scientific purposes over calf and adult serum due to more growth factors in the serum and less antibodies and toxins which match current scientific needs (Fang et al. 2017). Serum from other livestock is available, however only cattle, pigs, and poultry produce enough blood to support commercial production of serum Lee et al. 2023. Bovine serum is the lower cost option among animal-based serums that could provide an optimal culture medium (Arora, 2013). Synthetic alternatives to animal-based serum are available, however none of the alternatives have been able to fully compete with or replace FBS in many of its existing uses (Batish et al. 2022; Stout et al. 2022; Lee, 2024; Schenzle et al. 2025). Estimates of the size of the FBS industry have been growing; Jochems et al. (2002) estimated 500,000 L, Brindley et al. (2012) estimated 600,000 L, and Pecora et al. (2020) estimated 800,000 L. Current estimates suggest the overall FBS industry is worth over one billion dollars and could grow to well over 2 billion dollars in the next 5–10 years (Faizullabhoy 2023; Fetal Bovine Serum Market Size & Share *2024–2030*, 2024).

There is a market inefficiency in FBS production. Demand in the retail market is well developed and extensive Faizullabhoy 2023; Fetal Bovine Serum Market Size & Share *2024–2030*, 2024). Yet the cull cow market provides little incentive to increase the production of FBS. This has become especially problematic for FBS consumers expecting consistent supply of FBS. Furthermore, recent high cattle prices are causing fewer pregnant animals to be sent to slaughter. This raises the question of why the production and marketing of FBS has evolved as it has and whether there might be ethical ways of increasing the supply of FBS?

Previous research (Murray and Versteegen 2019) has described FBS production and marketing after the product has been processed, but this paper focuses more on packers, feedlots, and cow-calf producers. The paper is mostly interested in why the market provides little incentive to producers, but it also considers what could be done to increase supply.

Prices for FBS have been highly variable because of an inelastic supply (Siegel and Foster 2013). Given the importance of FBS, it seems useful to ask why the FBS market is so unresponsive. Despite its importance for research on human health, beef and dairy industries have no incentive to increase supply. As a result, feedlots sometimes pay to abort calves to avoid potential calving problems from pregnant heifers. This is despite Wiebe et al. (2023) finding that aborting the calf caused heifers to have a 0.18 kg lower average daily gain for the first 80 days. This resulted in 14.5 fewer kg on average and a loss of $58.88 in revenue assuming $4.06 per kg of beef. Similarly, Jim et al. (1991) found that therapeutically aborted heifers had lower average daily gains. Jim et al. (1991), however, found that aborted heifers had heavier carcasses than pregnant heifers. Since many slaughter cattle are sold based on carcass weight, the heavier carcasses of aborted heifers can provide incentive to abort the fetal calf.

Another potential source of FBS would be purchasing cows for slaughter that are sold as breeding animals. Past literature has only found small premiums for cows further along in their pregnancy, which supports that these animals are not being purchased to produce FBS. For example, Mitchell et al. (2018), Boyer et al. (2020c) for their November sale, and Boyer et al. (2020a) all reported premiums of $50–$60/head^1^ for heifers or cows seven months bred versus four months. Boyer et al. (2020c) reported a higher premium of $134/head for their May sale, but this may be partly due to the seven months bred heifers avoiding calving during summer heat. These studies are for breeding animals and so these premiums do not necessarily apply to animals destined for slaughter, however they indicate the potential to bid away breeding animals to use as a source of FBS.

Some authors have expressed ethical concerns revolving around the animal welfare implications of FBS production (Jochems et al. 2002; Gstraunthaler et al. 2013; Nielsen and Hawkes 2019; Versteegen et al. 2021). Proponents argue that significant legislation already protects animals’ welfare throughout the collection process and additionally upholds the standards for quality serum free from any contamination (Versteegen et al. 2021; International Serum Industry Association n.d.). Others argue that researchers may still have ethical concerns about animal welfare during the collection process even with current animal welfare protections, and that the risks of viruses and bacterial contamination, as well as recent price increases make it beneficial to keep searching for suitable serum alternatives (Jochems et al. 2002; Gstraunthaler et al. 2013; Nielsen and Hawkes 2019). In the short run at least, FBS is still a much-needed product while suitable serum alternatives are being developed (Fang et al. 2017; Lee et al. 2023).

## Materials and methods

Institutional animal care and use committee (IACUC) approval was not required as no animals were used or handled in this study. Informal interviews, budgets, and regressions are used to describe and quantify the industry for FBS, specifically the three main focuses of this paper: the marketing channel and production process, production budget, and market incentives. To describe the marketing channel and production process, industry employees were interviewed to obtain up to date information as a starting point for further research. Production budgets were used to determine revenue potential and industry costs. To identify existing market incentives and opportunity cost for producers of pregnant cows, least squares mean regression is used on replacement and slaughter cow auction price data.

### Production process and industry characteristics

To gather information about the production and marketing systems for FBS, responsible individuals at major meat and FBS processing firms were interviewed. Their responses characterized the production system and industry structure, as well as providing initial explanations from their perspective on the current system at the time of the interview. Due to the small number of people interviewed and the proprietary nature of the information provided, no formal statistical analysis is included.

Interviews took place remotely either via zoom call or over the phone in January of 2024. Interviews were kept short, between 30 min and an hour, with 4 individuals from some of the leading packing and serum-processing companies in the United States FBS supply chain. Given that beef packing and serum-processing are concentrated industries, a small number of individuals were interviewed as a very small number of firms are responsible for the vast majority of FBS collection and processing. Questions for each interview varied based on the job role that each person had relative to the serum industry. Individuals were chosen based on their proximity to serum production, specifically from the cattle processing side, and all held roles that were responsible for byproduct marketing or production operations. Each individual was speaking for their company; therefore, Internal Review Board (IRB) approval was not required.

These interviews included questions asking for expert opinion estimates including current prices for raw serum, quantities sold, and timelines for production. These numbers were used in the revenue calculations for the enterprise budget for FBS production, and to corroborate the exact sources of revenue and expenses incurred by the producer. Aside from interviews, prices of finished FBS were taken from ThermoFisher, a large retailer of serum (ThermoFisher Scientific, 2024b). Opinions expressed by industry individuals also contributed to our formation of the preliminary hypothesis of the paper as discussed earlier, that incentivized production is not taking place currently due to the industry’s perceptions of the public’s reaction to the animal welfare implications of FBS production. Kaminski et al. (2024) found that consumers placed high importance on animal welfare concern in the dairy industry, so the industry’s concern about animal welfare is supported by research.

### Auction cattle price comparison

Data on cull and pregnant cow prices were used in the enterprise budget as well as in determining price differences between culled cows and pregnant cow characteristics. Data containing auction prices of culled cows intended for slaughter and replacement bred cows were obtained from historical USDA AMS Livestock, Poultry, and Grain market news reports from Oklahoma City, Oklahoma and compiled by the Livestock Marketing Information Center (LMIC). The data contain monthly prices starting in April 2019 and extend to May 2024. Replacement bred cows are categorized by age, trimester of pregnancy, and grade (1, 1&2, and 2) as defined by the USDA grading (Feeder Cattle Grades and Standards n.d.) with 1 being the larger or more promising grade and then 1&2 and 2 having subsequently lower quality characteristics. The reported prices are averages across all lots sold at the day of sale, and some categories have no lots sold in a given week. Prices for bred cows are reported in dollars per head, while slaughter cow prices are reported in dollars per hundredweight (cwt). Slaughter cows are categorized by the USDA as low dressing lean, lean, boner, and breaker depending on their body composition score where breaker is the highest quality and body condition score, and low dressing lean is the lowest. For ease of comparison, slaughter cow prices are converted to dollars per head by assuming approximate cow weights of 1100 lbs., 1200lbs., and 1600 lbs., for lean/lean low dressing, breaker, and boning, respectively, and based on average weight reported for each category by the USDA AMS reports.

The analysis of variance model for the bred cows was


(1) $$\;P_{\mathrm{amgt}}^{B}=\;\mu_{0}+\tau_{a}+\gamma_{m}+\delta_{g}+\vartheta_{t}+\varepsilon_{\mathrm{amgt}}^{B}$$

Where $P_{\mathrm{amgt}\;}^{B}$ is the price in dollars per head of bred cows at age *a*, at trimester *m*, with grade *g*, in time period *t*, and *µ*_0_ is the grand mean. Next, $\tau_{a}\;$is a fixed effect for the age of the cows in the lot with age groups two to four, five to eight, and eight years plus, $\gamma_{m}$ is a fixed effect for the trimester of pregnancy, $\mathrm{and}\;\delta_{g}\;$is a fixed effect for the grade of the lot with grades one, one & two, or two.

The equation for the slaughter cows is specified similarly:


(2) $$\;P_{gt}^{s}=\;\mu_{0}+\delta_{g}+\vartheta_{t}+\varepsilon_{gt}^{s}$$

Where $P_{gt}^{s}$ is the price in dollars per head for a slaughter cow and $\delta_{g}\;$is a fixed effect for the grade of the lot with grades breaker, boning, lean, and lean low dressing. In both equations, $\vartheta_{t}$ is the year random effect with $\vartheta_{t}\;∼\mathrm{iid}\;N(0,\sigma_{\vartheta }^{2})$, and $\varepsilon_{it}∼\mathrm{iid}\;N(0,\sigma_{\varepsilon }^{2}).$ The threshold for significance will be *P* < 0.05.

## Results

### Production process and industry description

**Figure 1:**
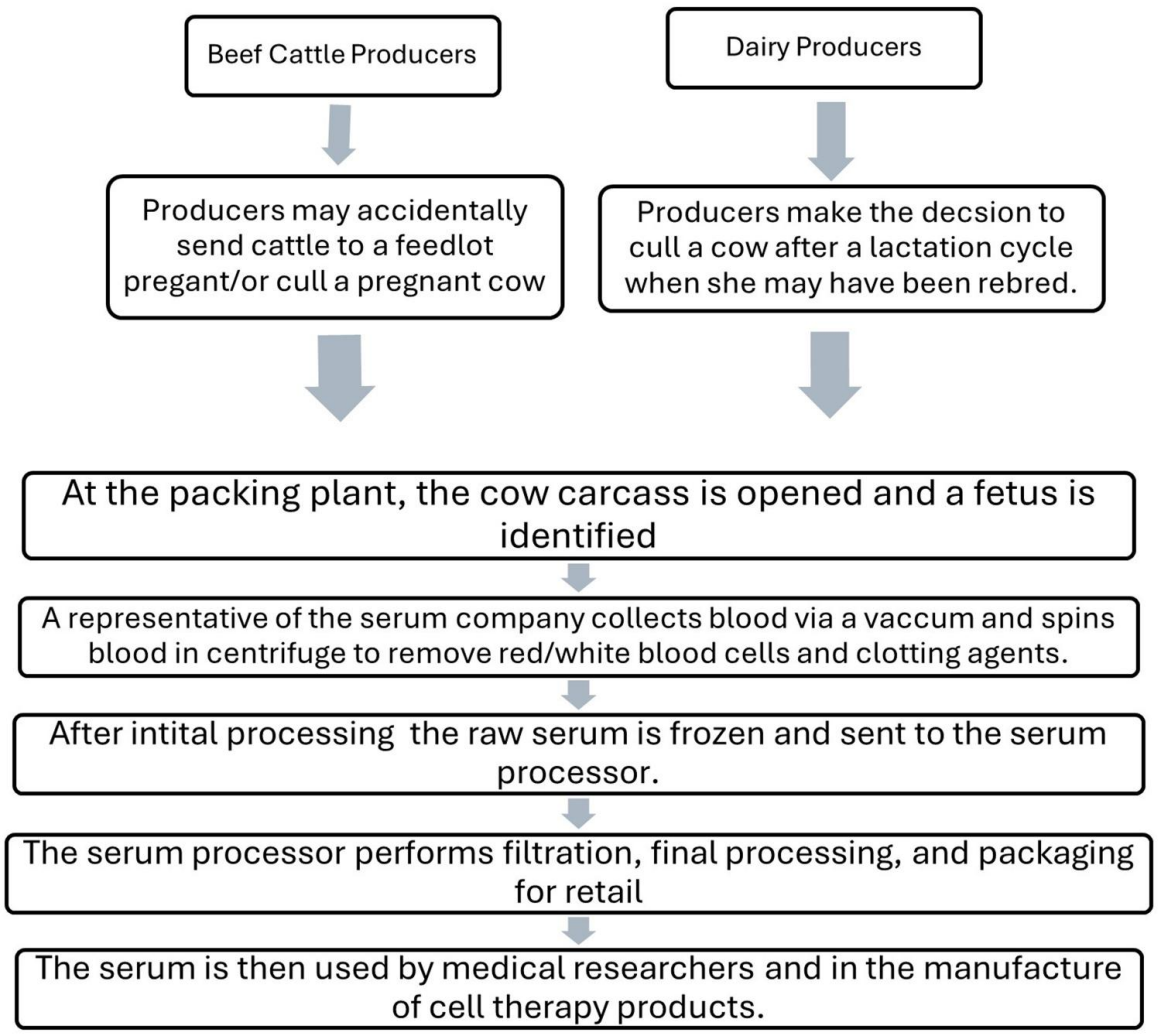
FBS Production process from producer to consumer.

A graphical description of the production and marketing channel for FBS is provided in Fig. 1. As Fig. 1 shows, there are three sources of pregnant animals: cull dairy cows, cull beef cows, and feedlot heifers. Dairy cows are culled at a younger age (Moreira et al. 2021) than beef cows (USDA 2021; Erol et al. 2024), but the beef cow herd is roughly three times that of the dairy herd (USDA 2025). The production system for dairy cows is such that culled dairy cows are often pregnant when culled. Beef cows may be culled because of difficulty rebreeding as well as other reasons (Raper & Biermacher, 2017) and thus beef cows are more often open when culled than are dairy cows. Pregnant fed heifers are not common. Fetuses are removed from the carcass and those over one month into gestation are held for serum collection. After removal, the fetal sack is opened from the placenta to reveal the “slunk.” Contracted representatives of the serum lab (or less often packing plant employees) collect all blood, which contains the serum, directly from the heart. This is commonly done using a bag that is hung above the calf that has a vacuum to make sure everything drains from the calf. The blood needs to be collected from the calf within 30 min of its extraction. The blood is stored in blood bags until it is centrifuged to remove red and white blood cells and clotting agents which is then considered raw serum. The raw serum is then stored in 1-L containers and frozen to ship to the lab for further processing. All serum processing must take place within 4 days and then it needs to be refrozen. The final processed FBS can be stored frozen for up to 5 years. Once the blood is collected, 52–57% remains as serum after the blood cells and other agents are removed.

FBS is a small part of packer income and its production is incidental. In addition, animal welfare concerns are also prominent among the reasons that FBS is treated as a byproduct. No major packer would want to be known for incentivizing the slaughter of pregnant cows. Packers treat FBS as a low-priority byproduct. The financial incentives are large enough that serum is collected rather than discarded. Note, however, that in a competitive market, the returns from FBS are passed on to the producers of cull cows. They are, however, passed on in the form of higher prices for all cull cows rather than just those that produce FBS and so they do not provide an incentive to provide cull pregnant cows. This creates a market inefficiency similar to the one that existed with fed cattle before grid pricing (Hogan et al. 2023) was introduced.

The quantity of FBS can vary substantially by plant. Areas with a higher concentration of dairy production produce more FBS. Cull cows from some beef herds are also known to be more likely to be pregnant. The frequency of positive identification also depends on the time of year. Spring and fall, which is typically calving season in beef production systems, is when the greatest volume of FBS is collected, as calves are closer to term. Producers interviewed reported 15–30 L of FBS is produced on average weekly at a larger plant.

Information on manufacturing, technology, and processes is mostly proprietary and specific to that manufacturer. Grading is determined by the individual product manufacturers as there is no governmental or private organization to standardize FBS grading. There is the International Serum Industry Association, which is a global organization, but their role is largely informational and assisting countries and final product producers in having a traceable product that meets standardized regulations regarding the country of origin. Two key measures of quality that are used are endotoxin and hemoglobin levels. Endotoxins, which are bacterial components, usually have values less than 1 Endotoxin Unit (EU)/ml in acceptable final serum. Values between 0.15 EU and 0.25 EU are common. For hemoglobin (leftover blood cells), the acceptable value for the highest grade is less than or equal to 15 mg/deciliter of serum. Companies may provide a certificate of analysis listing the endotoxin and hemoglobin levels in the FBS, however because blood is not fully defined, the effectiveness of serum cannot be guaranteed because it is unknown what the exact composition of serum is. Serum is offered in different grades specific to each retailer, the highest grade FBS is more refined usually resulting from 40 nanometer filtration where the composition of the FBS is almost fully known, from there the grading is based less on filtration and more so on the values of endotoxins and hemoglobin.

### Production budget

A production budget is developed for a packer selling to a serum processor. Revenue is the quantity of serum times the price received for the raw blood, and the major expense is the cost that is incurred by the packer for the weight of the fetus. While the fetus hide, after the blood is drained, is occasionally shipped to Japan or out of country where it is used to produce religious texts or other items for religious ceremonies, this is rare and so the fetus itself is assumed to have a value of zero.

At the retail level, pricing of FBS varies widely between countries, in the U.S. in 2024 it is approximately $800 per 500 mL as shown in Table 1, but Australia is up to $1400–$1500, compared to Canada or Brazil where it may only be $400–$500 (ThermoFischer Scientific 2024b), this is largely due to the diseases in the country, Australia does not import cows, so there is little to no concern of contamination. Prices of FBS have doubled in the last few years because of increased demand for more medical research and medical products requiring cell growth media.

**Table 1:** Retail prices ($/bottle) for fetal bovine serum obtained 23 January 2024.

	Grade		
Category/Unit	Value ($)	Premium ($)	Premium Plus ($)
*Heat active*			
* 500 mL*	492	700	836
* 50 mL*	62	87.50	104
* 10 × 50 mL*	582	826	986
*Heat inactive*			
* 500 mL*	526	784	894
* 50 mL*	66	93.50	112
* 10 × 50 mL*	622	884	1056

*Source.* ThermoFischer, 2024a.

Table 1 lists retail prices from ThermoFischer, one of the largest retail suppliers of FBS. These are prices of FBS available to consumers in the United States, and they may differ globally. The price of FBS varies depending on the grade required for the end use. Price also depends on quantity as it is shown that there are volume discounts. FBS that is heat inactive has been processed so that certain proteins from the immune system in the FBS are inactivated to avoid interactions in certain research (*Heat-Inactivated FBS (HI FBS)* n.d.). On average, retail returns for 1 L of serum based on the prices above would be approximately $2411.76.

In terms of revenue, the packer is paid per liter of blood. The price per liter of blood was reported by companies in interviews to range from $50/L- $200/L, however most recent prices range from $140/L-$143/L so $140 is used. Quantity of blood produced by an average calf can be calculated using the weight of a fetal calf as reported in Sorensen and Beverly (1978) and Moseley et al. (1929). A fetal calf is approximately 17% blood (Möllerberg et al., 1975), so a near term calf will yield approximately 7 L of blood.

Packers identified the weight of the calf as their only major cost. At cow processing plants the blood is collected by employees of the serum producing labs and not by the meat processor, therefore labor costs would not be accrued by the packer but instead by the serum retailer/processor. Similarly, costs associated with the collection equipment, initial storage, and transportation is the responsibility of the serum retailer/processor, so would not be an expense for the packer. The price of added weight due to the calf is from the recorded prices for boning and breaker cows in Oklahoma and averaged from 2022 to 2024 for a price of $2.22/kg. A pregnant cow would also be carrying additional weight from the placenta (including all membranes) as well as the amniotic and allantoic fluids. The placenta will weigh approximately 5 kg at the end of the pregnancy (Simões and Stilwell 2021). For a 23 kg calf, the placenta and fluids together were estimated at 10 kg. Weight gained during pregnancy other than the weight of the fetus and placenta is considered a sunk cost. As shown in Table 2, the budget only has one revenue source and one cost source.

**Table 2:** Net profits to produce fetal bovine serum assuming a 23-kg calf.

Item	Qty	Price ($)	Amount
*Revenue*			
* Blood*	7 L	$140/L	
* Total revenue*			$980
*Expenses*			
* The calf portion of the cow^†^*	23 kg	$2.22/kg	$51
* Placenta and fluids^‡^*	10 kg	$2.22/kg	$22.20
* Total expenses*			$73.20
*Net revenue per cow*			$906.80

*Note*. This budget assumes the packing plant sells the raw serum to a third party for final processing. Also, note that most fetal calves are smaller than 23 kg. Pecora et al. (2020) estimated that the industry average was 0.4 L of serum per fetal calf, which would be 0.73 L of blood.

† Note that arguments could be made for adding an additional small amount for packer profits on the weight.

‡ Cost of the placenta will be borne by the seller when a buyer pays by hot carcass weight.

Table 2 shows the revenue, expense, and derived approximate profits of FBS production. Based on the assumptions provided, there is an expected profit of approximately $908 per pregnant cow.

**Figure 2:**
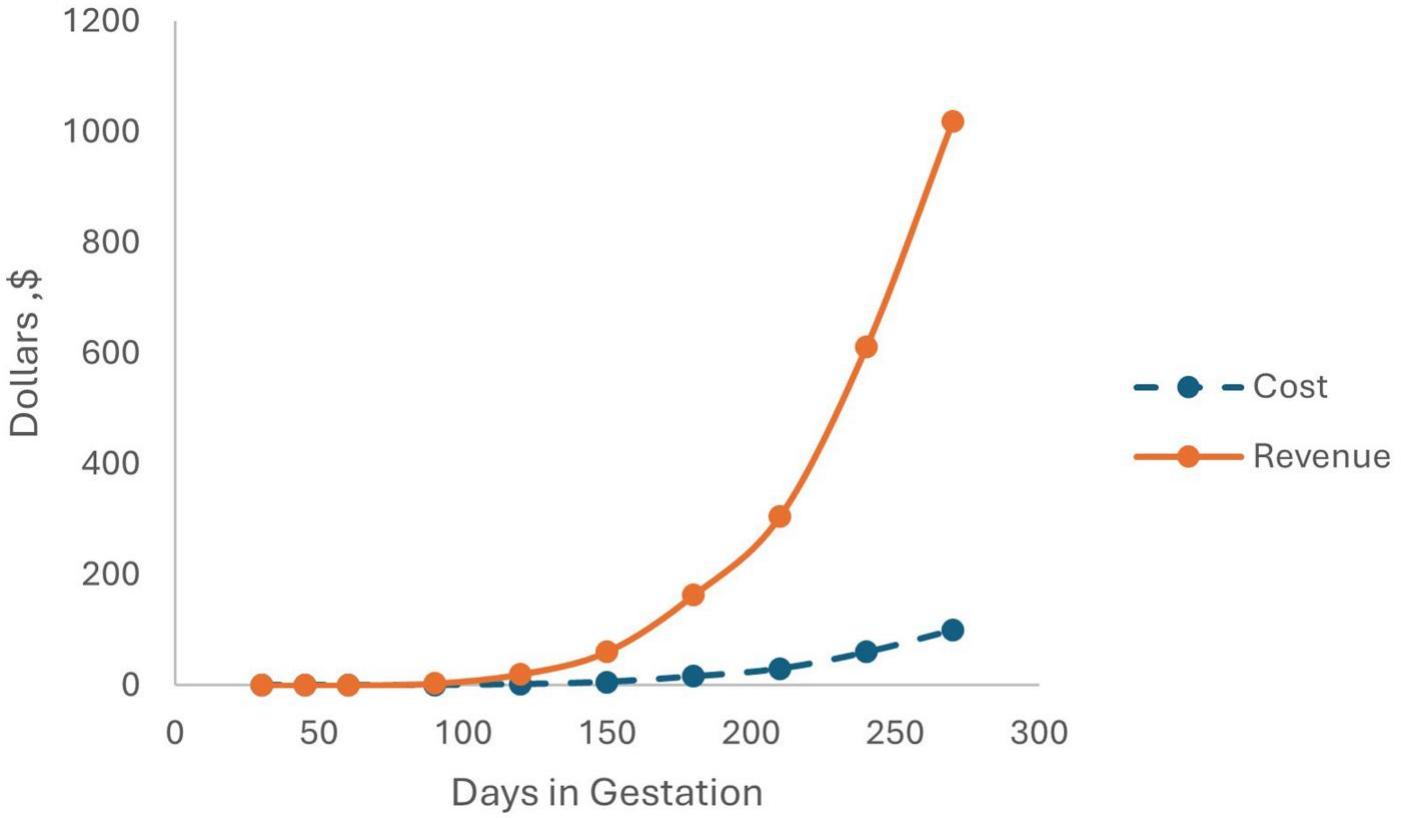
Revenue and costs of FBS production by months of gestation. Source: Interviews, Jochems et al. (2002), and Sorensen and Beverly (1978).

Figure 2 illustrates the change in profits throughout the duration of a cow’s pregnancy. The older the fetus, the more blood it produces. Figure 2 also illustrates how costs compare to revenue and the growth in profits as the fetus gets older. The cost curve is found by multiplying the weight of the fetus (Sorensen and Beverly 1978) and placenta (Simões and Stilwell 2021) at the given month by $2.22, the approximate cost per kg for cattle. To derive the revenue line, the quantity of blood is multiplied by the approximate price of $140/L.

### Characteristic comparison coefficients

Table 3 shows the coefficient estimates from equations (1) and (2). There is a higher premium for cows that are 7 to 9 months bred respective to cows only 1–3 months bred; however, the difference is only $56.47 and $21.64 from the 1 to 3 months bred to 4 to 6 months bred cows, respectively. Younger cows and cows between 5 and 8 years old had similar premiums with a difference of only $6, but there is a discount of $83.42 on average for a cow over 8 years old. Grade 1 replacement heifers had small premiums because they are deemed higher quality. Breaker graded slaughter cows showed higher premiums than the boning or lean slaughter cows, which was also as expected as they are considered higher quality. A breaker grade slaughter cow had a price of about $1,215 per head, while a near term replacement cow is only priced at $1,013 per head which is a difference of approximately $202. Given the results of table two that showed a positive profit opportunity of $908 for a replacement cow, this difference is even larger.

**Table 3:** Least squares means of bred and slaughter cow auction prices ($/head).

Characteristic	Estimate ($)	SE
*Replacement cows*		
* Months bred*		
* 1–3*	956^a^	8.33
* 4–6*	991^b^	6.25
* 7–9*	1013^c^	7.89
* Age of cow*		
* 2–4*	1018^a^	6.58
* 5–8*	1013^a^	5.85
* 8+*	929^b^	10.75
* Grade*		
* 1*	1192^a^	8.19
* 1&2*	963^b^	6.24
* 2*	805^c^	8.58
*Slaughter cows*		
* Grade*		
* Breaker*	1215^a^	0.32
* Boning*	957^b^	0.32
* Lean*	771^b^	0.32
* Lean low dressing*	688^c^	0.32

*Note*. Results with different letters assigned denote the means of each group are statistically different.

## Discussion

Given the results of our price analysis and budget estimates, there is a market failure in the FBS market. This is characterized by the estimated $908/calf (Table 2) in economic incentives in the market to produce more FBS that are being passed on to all producers rather than to the producers of pregnant animals. Retail prices for FBS are much higher, ranging from $984 per liter to nearly $2000 per liter, compared to the raw blood costs of only $140 per liter. The results in Table 3 show the difference between an early term pregnancy cow and a near term pregnancy cow is at most a premium of $56. An additional consideration is the $202 difference between a high-quality slaughter cow, and a 7–9 month bred cow. This shows even less evidence of incentivized production since selling an open cow would mean a potentially higher return, however there could be up to an $1110 ($908 from FBS + $202 from bred cow discount) incentive for a packer to purchase bred cows for slaughter. This result highlights that there could be the opportunity for revenue from the production of FBS which would incentivize packers to consider purchasing bred cows. This further supports the fact that there is little financial incentive to provide more FBS.

The current lack of incentives could be problematic for the future of FBS supply based on current short-term trends in the cattle markets. Current prices for calves are relatively high, which provides reverse incentives for any producer to send a pregnant cow to slaughter given that a live calf is so valuable. Even the estimated $1110 incentive would be less than what some calves are selling for in the 2026 market, but this may not be true in a period with higher calf crop number. Tight cattle supply and recent advances in sexed semen are reducing FBS supply. Sexed semen allows dairies to select the sex of the fetus with high accuracy (Seidel and DeJarnette 2022), so dairies use female semen and dairy sires only to produce enough replacement heifers and use Angus sires and male semen on other cows. This leads to fewer female dairy heifers and is expected to lower dairy cull rates and thus less FBS. Similarly, developments in dairy/beef crossbreeds are causing prices for crossbred dairy steers to be far higher than they have ever been (Berry 2021; Grote et al. 2026), so pregnant cull dairy cows are retained until the calf is delivered (Berry 2021). These recent trends mean a lower supply of FBS unless there is an increased incentive to produce fetal calves at slaughter.

There may be opportunities to modify current practices that could provide more FBS at low cost to beef producers. Current recommendations are for a 45- or 60-day beef cattle breeding season (Selk 2020; Johnson 2024) especially for more risk averse producers (Boyer et al. 2020b). At weaning, cows are pregnancy checked, and open cows are sold. It would be a low-cost practice to leave the bull in the pasture past 60 days and then cull cows that are not as far along in gestation (although ultrasound measures of gestation length are not as accurate as measuring open animals). At the feedlot level, feedlots often abort a fetus if a heifer is pregnant at arrival (Rademacher et al. 2015) (although not as often with today’s high cattle prices). This incurs additional expenses to the feedlot and can affect the overall daily gain for that heifer (Jim et al. 1991; Buhman et al. 2003, Wiebe et al. 2023). Feedlot producers may be able to capture some of the returns from fetal bovine serum if they chose not to immediately abort the fetus. An additional opportunity for feedlot producers could be breeding heifers or cull cows before being sent to auction, if an incentive is established for FBS production. There would be additional expenses such as the cost of AI breeding and estrus synchronization which is reported to be around $50 per head (Johnson and Jones 2008; Lima et al. 2010; Griffith et al. 2020). For that purpose, selecting sires with higher birth weights such as a show-calf sire could lead to a larger calf and larger quantities of serum. Other costs for feed and deductions for carcass grades may be negligible as studies have shown that even when fed the same diet as open heifers at a feed lot, carcasses from pregnant heifers had the same or higher quality grade as open heifers with just a slightly lower yield grade in some instances but not all (Jim et al. 1991; Kreikemeier and Unruh 1993; Bishop et al. 2003; Duarte et al. 2013). To maintain a consistent supply of FBS, an incentive for producers may be necessary given recent trends, and this could be a feasible option given that the incentive is higher than the breeding costs. Such incentives would need to come through a smaller packer, however, because larger packers are not going to take the public relations risk.

The results of this study are consistent with many other studies that assess related topics. Mitchell et al. (2018) found that the youngest cows had the largest premium relative to other ages which supports the results of the models in this paper. The same study again is consistent with our results that found the greatest premiums for cows in the third trimester of the pregnancy.

The regulation of FBS appears to be adequate, as several international agencies have regulations revolving around the safe import and export of raw and final FBS including the World Health Organization, the World Organization for Animal Health, European Medicines Agency, the U.S. Food and Drug Administration, and the World Trade Organization. The lack of established grading standards means that reputation is an important part of quality measurement. The importance of reputation may serve as a barrier to entry in serum processing, so market power in serum processing is possible.

## Conclusion

FBS is treated as a byproduct with currently no economic incentives to produce fetal calves. The demand for FBS is great enough that there could be an economic incentive to provide pregnant cows for the purpose of producing FBS. Fear of the public’s scrutiny of the FBS production process especially related to animal welfare, currently prevents market incentives from being passed on to producers. Specifically, the budget shows potential profits of $908 per cow that is found to have a 23-kg fetus, however there is only a maximum of $56.47 increase in premium for a cow that is further along in the pregnancy and would produce more serum. Returns to packers are low compared to revenue from retail, which would be approximately $2411.76 when taking an average of the prices for 1 L from Table 1. Due to the competition in the market, prices packers pay for a cow would reflect the expected revenue from FBS. This premium would be passed on to all producers and not necessarily gained by the producers that produced the FBS. Pressure to continue to produce FBS is going to need to come from users of FBS and from producers as packers are not the ones benefiting from its continued production.

FBS is valuable and an important ingredient in research on human health, yet the supply of fetal bovine serum has remained constant and inelastic like Siegel & Foster (2013) argued. Profits resulting from FBS production may be additionally valuable to small packing plants where increased production may be more feasible. The key to understanding why the price of FBS is high is understanding the arguments against changing the economic incentives that could lead to more FBS being produced.

Providing incentives to send pregnant animals to slaughter may require a change in societal values. Animal welfare and public relations concerns among packers limit the supply of the serum to being strictly incidental. While FBS revenues may still not be enough to entice the larger packers to take on the risks of furthering production, a small packer or integrated firm may be able to benefit from specializing in FBS collection.

## Footnotes

1 For Mitchell et al. (2018) and Boyer et al. (2020a) the premiums per head were calculated using the information reported in the paper while Boyer et al. (2020c) report a value of $54/head.

## Abbreviations

FBS: fetal bovine serum
LMIC: Livestock Market Information Center
